# Using Photovoice to Explore Students’ Experiences With a Hydroponic Shipping Container Farm

**DOI:** 10.1177/10538259241286496

**Published:** 2024-10-04

**Authors:** Gabrielle Edwards

**Affiliations:** 1 Department of Food and Nutrition, and Sport Science, 3570 University of Gothenburg, Gothenburg, Sweden; 2 Department of Curriculum and Pedagogy, 8166 The University of British Columbia, Vancouver, Canada

**Keywords:** experiential education, multimodal/multimedia, sustainability, environmental education

## Abstract

**Background:** Hydroponic shipping container farms (HSCFs) are an emerging tool being used in schools to educate and feed students. **Purpose:** This research explored students’ perceptions of HSCFs as an educational tool. **Methodology/Approach:** This research utilized a photovoice methodology whereby Canadian students aged 12–15 took photos of aspects of the HSCF, both positive and negative, that they found to be significant. A class presentation and discussion followed the photo taking process where students collaboratively identified common themes. **Findings/Conclusions:** This research found that students had difficulty engaging with the HSCF due, primarily, to the small size of the HSCF and design features that limited students’ ability to properly use and maintain the HSCF. The nature-like and high-tech appearance of the HSCF increased student engagement but there were also safety concerns highlighted by students in their photos and comments. **Implications:** The design of the HSCF appeared to be one that was built for efficiency and profit and the physical design did not encourage student usage. Significant redesign in consultation with teachers and students is needed if HSCFs are to be used effectively as an educational tool for students.

Current food systems are unsustainable and contributing significantly to several global challenges including climate change, poverty, biodiversity loss, and malnutrition (Foresight, 2011; Gladek et al., 2017). Additionally, there are public health concerns associated with current food systems. Although enough food is being produced to feed the population globally, dietary patterns have led to almost half of the population being either malnourished and experiencing hunger or micronutrient deficiencies, or obese and at risk of suffering from comorbidities associated with obesity (Swinburn et al., 2011; Tulchinsky, 2010). Therefore, major food systems changes are needed to address these issues. Although change is needed at all levels, choosing sustainable foods has been identified as the most effective consumer-side action that can be taken for positive food systems transformation (IPCC, 2022). Sustainable foods are foods produced in an economically, socially, culturally, and environmentally sustainable way and prioritize environmental and human health at all stages of the food supply chain (Garnett & Fischer, 2016; Willett et al., 2019). Eating sustainable foods has positive health benefits as these diets are low in red and processed meats and high in fruits, vegetables, and whole grains (Hedenus et al., 2014; Tilman & Clark, 2014). Therefore, consuming sustainable foods can contribute to a reduction in the environmental impact of food systems as well as positively affect individual health (Willett et al., 2019).

Due in part to the significant amount of time young people spend there, schools have been identified as key locations to incorporate and promote sustainable practices such as sustainable food consumption (UNESCO, 2014). Food education in schools has been identified as a tool that can help young people understand the impacts food systems have on environmental and human health; ultimately empowering young people to take a more informed and active approach to how they engage with food (Krause et al., 2018; Sadegholvad et al., 2017; Wilkins, 2005). Specifically, experiential learning opportunities related to food have been identified as effective strategies to positively impact students’ food choices (Charlton et al., 2021; Varman et al., 2021).

Experiential learning can be defined as “learning in real-life contexts that involves learners in doing tasks, solving problems, or conducting projects” (Knobloch, 2003, p. 26). Experiential learning opportunities related to food, such as cooking and gardening, have been identified as strategies to encourage sustainable and healthy eating behaviors among children. Several systematic reviews have highlighted experiential learning as an effective method to promote healthy eating habits among children in primary schools (Charlton et al., 2021; Dudley et al., 2015; Varman et al., 2021). These learning activities could include school gardens, cooking programs, school farms and, recently, hydroponic shipping container farms (HSCFs).

HSCFs are a relatively new agricultural innovation that are being used both in commercial settings as well as a tool in educational institutions to both educate and feed students (Wagner et al., 2020, 2021). HSCFs are shipping containers that have been renovated to contain a soilless hydroponic food production system. The shipping containers are modified to house a stable environment that is controlled by temperature regulators, LED lighting, humidity controls, and software that monitors growing conditions. HSCFs have the potential to reduce water use compared to conventional farming (Raviv & Lieth, 2008) and meet the demand for local produce (Wortman & Lovell, 2013). These units have begun to be marketed and sold to educational institutions by commercial distributers, such as *Freight Farms*, Growcer, FarmBox Foods, and Pure Greens (FarmBox Foods, n.d.; Freight Farms, n.d.; Growcer, n.d.b; Pure Greens, n.d.). One main advantage of utilizing these units in schools is, unlike other school-based methods of food production like school gardens, HSCFs can produce food year long, not only in the summer months when many schools are out of session. However, HSCFs are costly being over $200,000 CAD to purchase (Growcer, n.d.a) with additional annual costs of between $8,000 and $16,500 USD ($10,045—$20,718 USD adjusted for inflation) to operate (Hicks, 2017). Despite their potential, there have been limited studies investigating HSCFs in an educational setting (Stapleton & Meier, 2022; Wagner et al., 2020) and no studies, to the author's knowledge, that investigate middle or high school students’ experiences with HSCFs. Students are often excluded from research related to school food (Asada et al., 2017; Kumar et al., 2017; Linton et al., 2014). This is problematic as, if students voices are not included in the research process, initiatives may be designed in a way that reduces student engagement. As students are the targets of initiatives related to school food, including their voices in research can lead to increased engagement and better success of these initiatives (Reich et al., 2015). Consequently, understanding how students engage with HSCFs is a major research gap. Therefore, this research aimed to highlight the voices of students and answer the research question “using photovoice, what are students’ experiences with an experiential food education program utilizing an HSCF?”

## Methods

This paper represents some of the results from a PhD dissertation (see Edwards, 2023). Ethics approval for this study was obtained from The University of British Columbia [H21-00961]. The photovoice project was part of a larger, year-long case study investigating participants’ experiences with a HSCF in an educational setting. Therefore, although this paper focuses on students’ voices as represented through photovoice, the results are situated within the context of the larger case study which included over 11 hr of interview audio and over 160 hr of naturalistic observations within the school environment. The larger study included the voices of teachers, school administrators, community partners, and project funders in addition to students. Future papers will include results from other parts of the study specifically related to learning expectations and outcomes of the HSCF program.

Although the focus of this paper was on students’ experiences as expressed through photovoice, not a critique of the photovoice methodology itself, it is important to note the effectiveness of this methodology in highlighting student voices. In other parts of the research project students engaged in several semistructured interviews exploring their perceptions of the HSCF program. The photovoice results have been highlighted separately from those findings as allowing students to utilize photography revealed distinct findings that were not discussed in the interview portion of the research project. Specifically, photovoice highlighted many aspects of the physical HSCF space that were not mentioned in other parts of the research project. Therefore, the use of photovoice expanded students’ ability to communicate their perceptions regarding the HSCF and added valuable data to the research project. The distinct findings that photovoice revealed shows the importance of including varied methodologies when including young people in research as different mediums can allow for more diverse responses that would otherwise be missed.

### Researcher Positionality

The researcher acknowledges that their background and experiences may have affected data collection and interpretation of results due to researcher bias. The researcher's background as a food literacy educator, experiences as a PhD student, and previous world travels have influenced the way they view food literacy education. These experiences have led the researcher to have strong beliefs regarding the need for transformation towards more sustainable food systems and the role everyone must play in that transformation. The choice of methodology and interpretation of research findings were influenced by these worldviews and the belief that everyone should be equipped with the tools and knowledge to be empowered to take an active and engaged role in food systems transformation.

The researcher had no previous experience with HSCFs prior to this research as HSCFs are still a new tool being used in educational settings. Therefore, the researcher held no preconceived ideas or expectations about HSCFs as an educational tool before conducting this research and all findings emerged out the researcher's interpretation of the research findings themselves.

This research was conducted through the lens of the social constructivist research paradigm. This paradigm emphasizes the importance of lived experience and understanding research from the perspective of participants. This paradigm assumes not one, but multiple realities (Creswell, 2014). Therefore, the findings presented represent one possible interpretation of participants’ experiences and are based on the researcher's interpretation as a white, heterosexual, woman who has experience in sustainable food systems education as an educator. Throughout the research process the researcher sought to be reflexive in how their life experiences and privileges may unintentionally be informing the research process (Dodgson, 2019).

### Research Setting

The photovoice project was conducted with a grade 9 class (students aged 12 to 15) at a Middle School located in a large population center in British Columbia, Canada. In 2019, this school received money from a Canadian charity to purchase and install an HSCF on the school property. The funding received also covered the expenses of running the HSCF for 5 years. In total the funding is estimated at over $250,000 CAD. Although the HSCF was installed on the school property in fall 2020, it was not able to be fully utilized until the 2021–2022 school year due to the COVID-19 pandemic. Therefore, the year of this study was the first year the HSCF was fully utilized as an educational tool within the school. The purpose of the HSCF was both to feed students at the Middle School and to educate students about food systems.

The main way that the HSCF was utilized as an educational tool was involving students from a grade 9 environmental studies class. This class became colloquially known as “the farm class” as most of the class time was spent running the HSCF. The class had a purposefully small size of eight students to ensure all students in the class were able to have access to experiential learning time in the HSCF.

Although the educational component of the HSCF program is not the focus of the paper, it is important to briefly discuss the school's approach to education utilizing the HSCF. The focus of the environmental studies class was to take responsibility in running the HSCF, which included activities such as planting, harvesting, cleaning, and maintaining the HSCF. The class itself did not have a clear curriculum as the teacher wanted student's passions and interests to drive class content. However, due to the large amount of time required to simply run the HSCF, the majority of the class was spent on day-to-day HSCF operations with little time available for focusing on learning content.

Data collection for the larger research project occurred from November 2021 until June 2022, the photovoice project occurred between January 2022 and March 2022. In this way, students involved in the project were able to have some months working in the HSCF before being asked for their thoughts on the HSCF program.

### Study Design: Photovoice

This research utilized a photovoice methodology. Photovoice is a participatory research methodology whereby participants engage in research through taking photos. It is a community-based research method that supports participants in examining strengths and weaknesses in their settings (Catalani & Minkler, 2010; Hergenrather et al., 2009; Sutton-Brown, 2014; Wang & Burris, 1997). The photographs taken could have broader emotional, social, and political meaning beyond the individual participants’ perspectives (Wang, 1999). Photovoice is seen as a tool that can increase accessibility and participation as it does not solely rely on verbal forms of communication (Cluley, 2017). Photovoice methodology can be a useful methodology to use with young people and students as it can enhance self-esteem and community engagement. In relation to health, well-being, and food, this methodology has been shown to be effective when working with young people (Findholt et al., 2011; Heidelberger & Smith, 2015; Lam et al., 2019; Spencer et al., 2019; Strack et al., 2004). Photovoice has been used to study the food environment from a community perspective (Belon et al., 2016; Díez et al., 2017; Gravina et al., 2020; Heidelberger & Smith, 2015; Ronzi et al., 2016) as well as to study the food environment within schools (Spencer et al., 2019). Research suggests that using photography in data collection may be a more effective way of engaging young people in research compared to using other methods alone (Lambert et al., 2013), promote leadership (Findholt et al., 2011), and increase young people's trust in the researcher (Clark, 1999).

However, photovoice is not without its limitations and there have been concerns raised about the degree to which participants are included in research (Evans-Agnew & Rosemberg, 2016), an overemphasis placed on the effectiveness of photovoice as a methodology rather than the outcomes for the study population (Bender et al., 2017; Drew et al., 2010), and data ownership (Golden, 2020) among others. This research sought to overcome some of these limitations by engaging in a participatory thematic analysis process that ensured participant voices were accurately represented and placing the focus of the research on how photovoice can positively influence HSCF programming rather than the effectiveness of the methodology (see analytic strategies section of paper). Despite its limitations and critiques, when conducted well, photovoice remains a useful and accessible methodology for engaging young people in research. Therefore, this research employed a photovoice methodology to include the perspective of student participants.

### Participants

The participants in this research were students in a grade 9 environmental science class. All students in the class were invited to participate and all eight students consented to participate in the research. No participants dropped out of the study; however, one participant missed the discussion session due to personal circumstances. That student later provided photographs and written comments regarding the photos which were taken into consideration during group discussions.

### Procedures

This study followed the photovoice procedures outlined by Wang (2006). The researcher was assisted by the lead HSCF teacher. In January 2022, the eight students who were part of the farm class were invited to take part in a photovoice project regarding their experiences with the HSCF. Two meetings were held with the students regarding the project. During the first meeting, the researcher gave a presentation to the students about what photovoice is, the purpose of the methodology, and some general information about photography. The researcher also went through the whole photovoice process with the students utilizing a past photovoice project as an example.

The students were then given three questions to consider:

- What they liked about the HSCF,
- What they disliked about the HSCF,
- What they would change about the HSCF.

These prompts were chosen to help students consider aspects of the HSCF that were working and ways the HSCF could be improved if there was an opportunity. The questions did not focus on education specifically as this study was exploring participants’ experiences with the HSCF program in general and did not want to limit participants’ responses to educational outcomes alone. It was emphasized to students how their feedback was important in shaping what HSCFs could look like in other educational settings. There were plans to share the students’ feedback with school administrators and project funders to inform the design of future HSCF units and programs located in educational settings. As this HSCF was one of a very small number of HSCFs located at educational settings in Canada or even North America, consulting students and other stakeholders about their experiences and using that information to shape the design of future HSCFs is critical.

Students were then given 3 weeks to take photos of elements that, from their perspectives, answered these three questions. During these 3 weeks the researcher and lead HSCF teacher checked in with students once a week to inquire about the project and offer guidance if needed by the students. All participants used their own mobile phones to take photographs. The photographs were then shared with the researcher using a secure file exchange system.

### Analytic Strategy

At the end of the 3 weeks, a participatory process of analysis occurred following the procedure outlined by Wang and Burris (1997). This analytic strategy was composed of three elements: selection, contextualization, and coding. First, each student was given time to review their photographs and select ones to be used for discussion. Second, they identified the significance of the chosen photographs. The students than prepared a brief PowerPoint presentation that included their photos that they presented to the class. Students contextualizing the photos with additional thoughts/comments that explained why they chose to take the photos they selected. Essentially, the students told the story of their selected photos and this contextualization gave meaning to the photos they selected. Third, these presentations were followed by a whole class discussion to identify common themes that emerged within the class. The researcher facilitated this discussion and, when needed, offered prompting questions such as “why did you take this photo?,” or “tell me the story of your photo.” In this way, the group discussion acted as a participatory thematic analysis where participants sorted the photos into common themes. This initial group identification of themes ensured that participants’ voices were accurately captured and acted as a type of member checking that enhanced the reliability of the study. All photograph presentations and the preceding discussions were recorded using a recording device. The audio recording was then transcribed by the researcher.

The identification of broad themes emerged out of this collective discussion and the themes were further developed by the researcher using Braun and Clarke's (2006) method for thematic analysis and the software NVivo. The coding process included the analysis of 24 photographs, the photographs’ accompanying texts, the audio transcript of the photovoice discussion, and was situated within the context of the researcher's understanding of the school environment as developed through naturalistic observations.

As action is an important part of photovoice methodology, students’ comments and concerns were communicated to school administrators through the class teacher. It was originally proposed that the students would present their finding themselves to school administration and project funders, however, due to some logistical issues this option was not possible.

## Results

The research data emerged through the photographs taken by students, the accompanying texts, and ongoing observations and conversations within the school environment over the course of the 2021–2022 school year. Three main themes emerged out of this project and are described below.

### Theme 1: Physical Design of the HSCF

**Figure 1.**
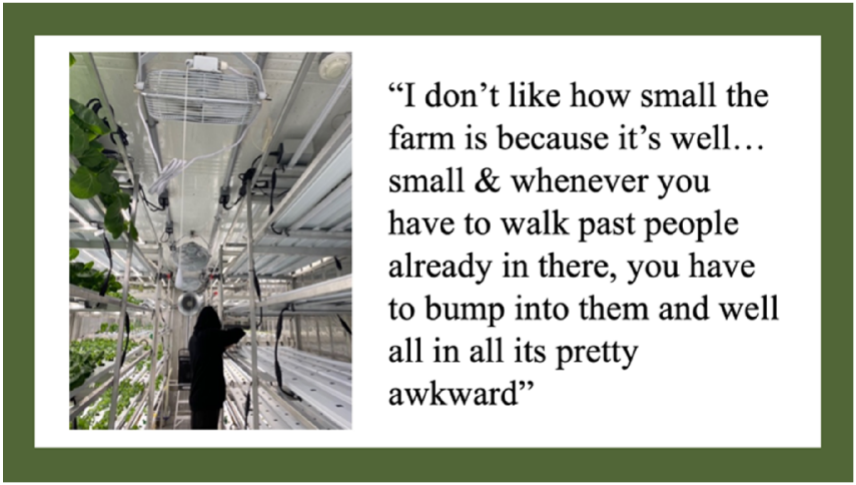
Photovoice artifact—theme 1: physical design of the HSCF.

In their photovoice presentations and the discussion that followed, students discussed how the design and infrastructure of the HSCF negatively impacted their experience working in the HSCF. They highlighted how the interior of the HSCF was not designed in a way that would accommodate large numbers of students. When the research participants worked in the HSCF, they were not all able to be inside at the same time. Only two or three students could comfortably work in the HSCF at one time. Even when there were few students in the HSCF, they still had difficulty effectively working in the HSCF due to lack of space. Therefore, the farm class required two adult figures to run effectively: the lead HSCF teacher as well as the researcher who aided the class as a teacher volunteer when required. The lead HSCF teacher would take half the students, and the researcher would supervise the other half. One half of the class would work in the HSCF while the other half engaged in classroom learning or other activities such as picking up garbage on the school grounds. Figure 1 highlights one student’s thoughts and photo related to the limited space within the HSCF.

HSCF = hydroponic shipping container farm.

**Figure 2.**
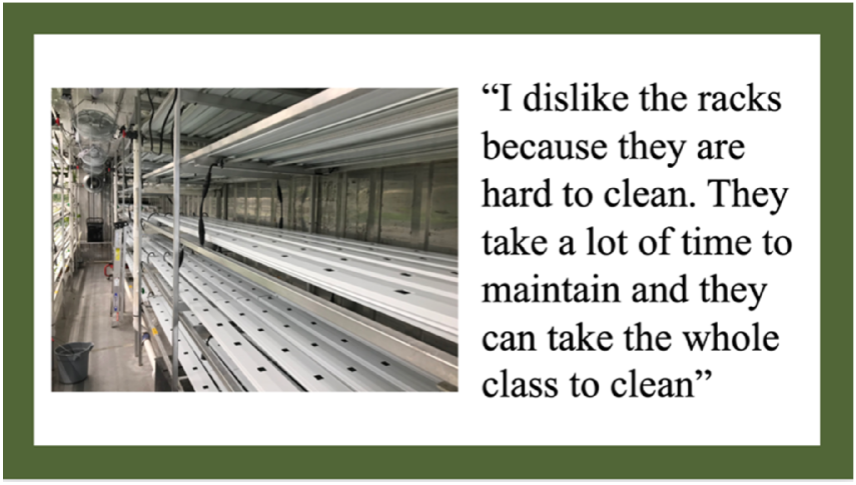
Photovoice artifact—theme 1: physical design of the HSCF.

In addition to students disliking the cramped space inside the HSCF, students also commented on how the HSCF's design made it difficult to perform HSCF tasks such as cleaning. They discussed how they could not reach all areas of the HSCF due to its cramped design which led some areas to become overgrown with algae due to students not being able to access those areas of the HSCF. Even the lead HSCF teacher commented on her inability to reach parts of the HSCF, especially the back corners. Figure 2 indicates one student's photo and comments regarding the design of the HSCF and how it was difficult to clean.

HSCF = hydroponic shipping container farm.

Table 1 includes further examples of the students’ thoughts regarding the physical design of the HSCF.

**Table 1.** Sample Student Responses Theme 1: Physical Design of the HSCF.

Theme	Sample participant response
HSCF design	“There's not much space to work … it's really small”
	“The farm seems kinda cramped and claustrophobic at times”
	“There's not enough space”
	“The farm is a little too small for how many people have to work in it sometimes. I feel like an expansion would be worth it because we can grow more vegetables at a time and also have a more comfortable working area”
	“I do not like the depth in which the trays stretch because even with a ladder it is quite difficult to reach the back and clean the trays”
	“Trying to get to the very back corners is basically impossible”

HSCF = hydroponic shipping container farm.

### Theme 2: Appearance of the HSCF

**Figure 3.**
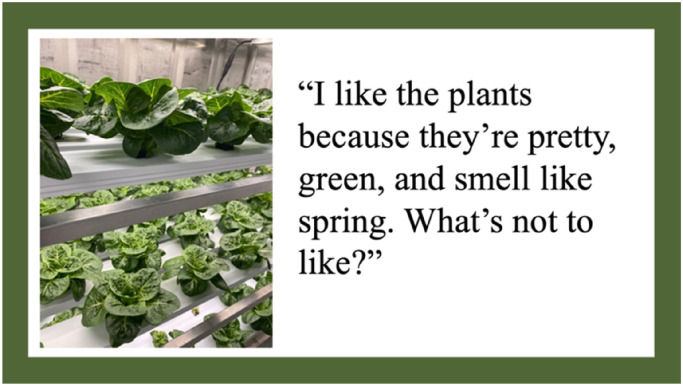
Photovoice artifact—theme 2: appearance of the HSCF.

When discussing aspects of the HSCF that they enjoyed, students indicated they enjoyed the appearance and aesthetic nature of the HSCF interior. There were two aspects of the appearance of the HSCF that students indicated they liked: the green and nature-like aspects of the HSCF, and the high-tech characteristics of the HSCF. Students enjoyed the fresh, green interior of the HSCF, and this pleasing aesthetic increased their enjoyment of the time they spent in the HSCF. At the same time, students also indicated that they enjoyed the high-tech and futuristic appearance of the HSCF's interior. In this way, the student's enjoyment of the HSCF's interior was in conflict: on one hand, they enjoyed the naturalistic features of the HSCF while on the other appreciating the feeling of being in a sterile environment quite disconnected from nature. Figure 3 represents one student's photo and thoughts regarding the interior appearance of the HSCF.

HSCF = hydroponic shipping container farm.

**Figure 4.**
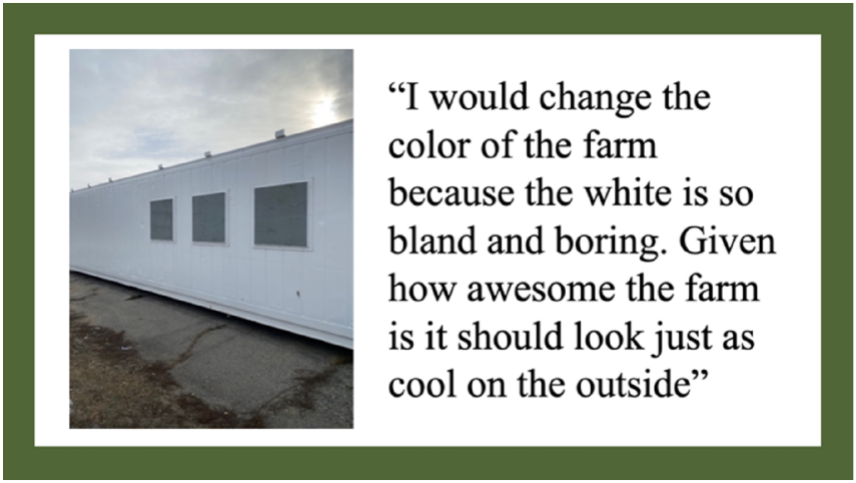
Photovoice artifact—theme 2: appearance of the HSCF.

Though students indicated that they enjoyed the internal appearance of the HSCF, they also discussed how they disliked the appearance of the outside of the HSCF. Specifically, they thought a plain white box with windows had little visual appeal. This dissatisfaction is demonstrated in one student's photo and comments (Figure 4):

HSCF = hydroponic shipping container farm.

Although the HSCF was already quite visible in the community due to media exposure and word of mouth, painting the exterior of the HSCF would enhance the visual appearance of the HSCF and make it more attractive more people walking or driving by the school. As the students were proud that they were participants in the HSCF program, they wanted the HSCF to be more visible and aesthetically appealing from the outside. There were plans at the beginning of the school year to have students paint a mural on the outside of the HSCF as well as the words to a local Indigenous song. The students even wrote a letter to a local paint shop and had paint donated for painting a mural; however, the class did not end up painting the mural in the 2021–2022 school year. It is possible that students in the future years may paint the HSCF. Table 2 includes further examples of how students described the appearance of both the interior and exterior of the HSCF.

**Table 2.** Sample Student Responses Theme 2: Appearance of the HSCF.

Theme	Sample participant response
Appearance of HSCF	“When it looks green and is all ready to be harvested it looks really pretty”
	“I really like how sciency the farm looks … it really looks like something out of Star Trek or Battlestar Galactica”
	“Here it's more technology stuff and I really like technology. Which is another reason why I like this farm”
	“I feel like I’m walking in a spaceship”
	“Inside the farm is really nice … I feel calm when I’m in the farm”
	“I don’t like how plain the outside [of the farm] is”

HSCF = hydroponic shipping container farm.

### Theme 3: Safety of the HSCF

While students did not specifically indicate that they felt unsafe while working in the HSCF, the photos, comments, and class discussion illuminated several possible safety concerns. These safety concerns had to do with the design of the HSCF itself. Students indicated that the low placement of the fans on the roof of the HSCF made them feel as if their hair could get caught in the fans while working in some areas of the HSCF. When students were required to reach the upper racks or back sections of the HSCF, they stood on ladders and often hit their heads of the fans. Although the fans were always turned off when students were working in those areas, the students’ safety concerns remained.

**Figure 5.**
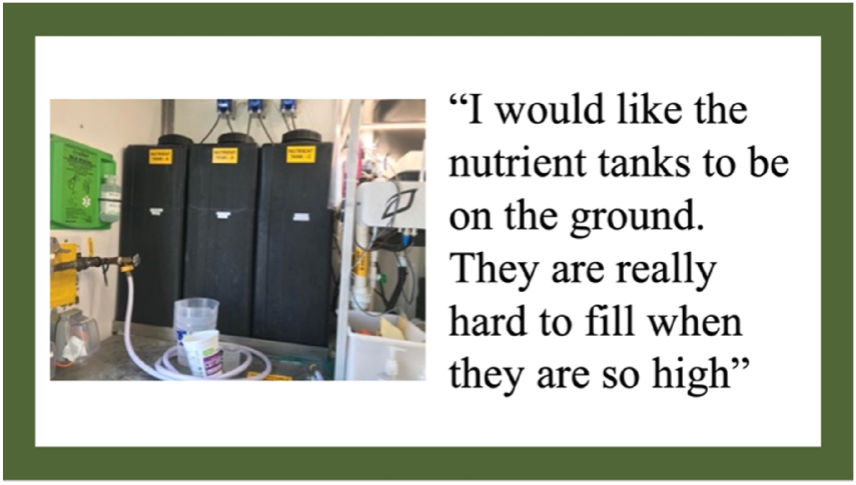
Photovoice artifact—theme 3: safety of the HSCF.

In addition to safety concerns regarding the fans in the HSCF, students also expressed concerns about some of the day-to-day tasks associated with operating the HSCF. Specifically, students highlighted concerns associated with the task of refilling the nutrient and PH buffer tanks in the HSCF. These tanks were placed on a shelf and were too heavy to move. Additionally, the containers given to the school with refill liquids were also very large and too heavy for students or the lead HSCF teacher to lift safely. As one of the containers contained acid, refilling this specific container presented a perceived safety risk for whoever refilled that container. Although the actual danger of the acid was minimal and would not cause harm to the students should they interact with it, the perceived safety risk was real. Figure 5 represents one student's photo and comments regarding the safety of the HSCF.

HSCF = hydroponic shipping container farm.

Table 3 provides additional examples of the students’ thoughts regarding the safety of the HSCF.

**Table 3.** Sample Student Responses Theme 3: Safety of the HSCF.

Theme	Sample participant response
HSCF safety	“The fans are too low. People who are very tall often hit their heads on the fans”
	“The fans, how they’re placed. When we’re working someone could get their hair or clothes stuck”

HSCF = hydroponic shipping container farm.

## Discussion

This research aimed to explore students’ experiences with, and perception of, a HSCF as a tool for food education. This was explored through a photovoice project that allowed students to express themselves through a visual media. Through selection and presentation of photos, contextualization, and group thematic analysis, students offered a critical perspective about their experiences working in the HSCF.

The first theme highlighted how students had difficulty engaging in HSCF farm tasks or learning opportunities due to the poor physical design of the HSCF. Students unanimously agreed that they disliked the small size of the HSCF. They indicated that it was too small and poorly designed which made it difficult for multiple students to work in the HSCF at one time. These concerns suggest that the HSCF at the Middle School was not designed in a way to be an effective educational tool. This concept was not discussed in previous studies exploring HSCFs in educational settings (Stapleton & Meier, 2022; Wagner et al., 2020). However, Stapleton and Meier's (2022) study which followed undergraduate student's involvement in a HSCF over the course of a school year indicated that only 12 students were consistently involved in the project. This confirms this study's findings that HSCFs are not able to accommodate large numbers of students in learning opportunities. Although HSCF have large year-round production potential (Wortman & Lovell, 2013), their feasibility as an educational tool appears to be limited. It is not cost effective for an educational institution to spend over $200,000 CAD on an HSCF for educational purposes if only a very small number of students will have the opportunity to be involved.

The second theme indicated students enjoyed the nature-like and high-tech aspects of the interior of the HSCF but disliked the plainness of the outside of the HSCF. The duality in terms of what about the appearance of the interior of the HSCF students enjoyed was striking. Some students indicated the green and nature-like appearance of the HSCF to be what they found enjoyable while others indicated the high-tech and apart from nature aspect of the HSCF was what they enjoyed. The students’ observations regarding the aesthetically pleasing look of the HSCF could be an indication that spending time in the HSCF could have been providing the students with therapeutic benefits. This finding would be in line with Stapleton and Meier's (2022) study which indicated that students derived mental health benefits from spending time inside a HSCF. Several other studies have confirmed the mental health benefits of spending time with green plants indoors (Bahamonde, 2019; McCormick, 2017; Mcsweeney et al., 2015). Students’ comments about the high-tech and futuristic appearance of the HSCF indicates that this technological aspect could increase student interest in working and learning in a HSCF. This is in line with the findings of many other studies that show technology increases student engagement, self-direction, and learning (Parsons & Taylor, 2011; Rashid & Asghar, 2016).

The third theme indicated that the HSCF at the Middle School may have had significant safety concerns regarding the internal design of the HSCF. Students expressed concern about getting their hair caught in fans, difficulty accessing all areas of the HSCF, and perceived danger from handling chemicals. Some of these concerns, such as worries about chemical burns, could be managed through incorporating training and use of personal protective equipment into the HSCF educational program. Other concerns, however, such as parts of the HSCF being unreachable and the placement of the fans, would be challenging to mitigate due to the design of the HSCF. These safety concerns appear to be unique to indoor forms of growing food like HSCFs compared to other experiential learning opportunities related to food. For example, school gardens would not have the same safety concerns as they are most often located outdoors so would not have safety concerns related to lack of space or placement of fans.

Regardless of the real or perceived safety threats the HSCF posed, if students do not feel safe in HSCF units, they may not want to participate in HSCF educational programming. Moreover, if an accident was to occur in an HSCF, Occupational Health and Safety may deem the HSCF as unsafe which could lead to the HSCF being shut down. Therefore, the safety concerns expressed by the students need to be addressed. Companies designing and selling HSCFs to be used in educational institutions need to be aware of design differences that are needed between using the units commercially and in educational settings. Consulting with schools, teachers, and students about the optimal design for a HSCF in a school setting is needed to mitigate safety concerns when having students work in the units.

Of significance was one aspect that students did not highlight in their photovoice projects. Students did not indicate that consuming the produce grown in the HSCF was an aspect they found particularly striking or enjoyable. This finding is of particular significance. Although every student in the class had the opportunity to consume produce or take produce home at any time, students rarely took advantage of this opportunity. In fact, some of the students in the class chose to never consume or take the produce home. As one of the purposes of the HSCF was to feed students, it is concerning that the students working in the HSCF did not want to consume the produce grown there. This highlights the importance of consulting with students when designing educational and school food related initiatives.

### Implications for Educators and HSCF Vendors

This photovoice project revealed that the design of the HSCF at the Middle School investigated in this study may not be suitable or safe for use in a school environment. Although finances were not the focus of this research paper, it is critical to note that it is not feasible for a school to invest over $200,000 in a tool that is only able to engage three to four students at a time. Although there was interest from other teachers outside of the farm class to bring their students into the HSCF, there was not enough physical space in the HSCF to accommodate all interested students. This challenge is likely unique to HSCFs due to their design. Because of the unit's inability to deliver hands-on learning opportunities to very many students, the educational potential of these units in their current form is low.

If HSCFs are to be marketed and sold to educational institutions, vendors producing these units should consult with schools, teachers, and students to determine a functional and safe design for a HSCF. The current design of the HSCF at the Middle School had elements that favored efficiency and productivity but detracted from the learning potential of the HSCF. For example, the cramped space in the HSCF that students discussed made it difficult to conduct classroom learning but would be ideal if the goal was to have one or two employees operating the HSCF. Therefore, HSCF manufacturers advertising and selling their products to educational institutions should work with schools, educators, and students to design HSCFs that are able to be effectively used in educational settings as the specific unit investigated in this study only allowed for a few students to participate in learning activities. Additionally, there is a risk of HSCFs placing a huge financial burden on schools that acquire them if they are not designed in cooperation with the educational institutions who are the intended end users of the HSCFs. Overall, this study concludes that the HSCF unit the Middle School received had elements of its design that detracted from its potential to achieve the school's goals in relation to the HSCF program. This points to a possible discrepancy between the current HSCF design and what is needed if an HSCF is to be used effectively in educational settings. These results also highlight the importance of including educators and students in the HSCF design process.

### Study Limitations

One limitation to this research is the small sample size of eight students. However, due to the emerging nature of HSCFs within school environments, there was a limited total sample size available. A second limitation is that this study only investigated one specific type of HSCF design. Not all HSCFs are alike and there are multiple designs being offered on the market that vary significantly in terms of space, internal set up of farm, lighting, cost, and more. Therefore, other HSCF designs may be better suited to educational settings, yet this study was only able to comment on one of the many possible designs.

A further limitation was the COVID-19 pandemic. The pandemic not only resulted in some students not attending all HSCF classes, but also limited the ability of the school and HSCF teacher to focus on planned HSCF activities and goals. Therefore, the experiences of the students as represented in this paper were within the context of an HSCF program that was disrupted several times throughout the year due to changing local public health orders.

### Potential for Future Research

HSCFs and other high-tech tools used for food education is an area that requires further research to understand their educational potential as well as possible limitations. This is especially true given the increasing global focus on the importance of technology in agriculture (see FAO, 2022) and the possible increased focus on technology in food education that may result from this shift. Further research is needed to confirm if the concerns indicated by students in this study are echoed by students in other schools using HSCFs as well if these concerns persist with other types of HSCF designs. Additional research is also needed to assess the cost-effectiveness of HSCFs as educational tools, the long-term sustainability of HSCFs in educational settings, the relationship between food education funders and educational institutions, and the overall educational potential of HSCFs in schools.

## Conclusion

Students’ voices indicated that the HSCF at their Middle School was not well designed to be used as an educational tool. There were limited opportunities for student engagement due to the small size of the HSCF and students found it difficult to work in all areas of the HSCF due to a cramped design. Additionally, students had safety concerns with the design of the HSCF. However, both the high-tech appearance and the aesthetics of the produce in the HSCF were aspects of the HSCF that students particularly enjoyed. The HSCF model at the Middle School appeared to have elements that promoted efficiency and profitability, rather than being an engaging and productive space for students. If HSCFs are to be used as an educational tool in schools, the units should be designed differently than the unit investigated in this study and, ideally, school stakeholders such as teachers, administrators, and students should be incorporated in the design process to ensure the unit is optimally designed for a specific school's needs and goals.
